# The prognostic impact of surufatinib for the treatment of advanced pancreatic ductal adenocarcinoma: a single center real-world retrospective study

**DOI:** 10.3389/fonc.2025.1574934

**Published:** 2025-05-20

**Authors:** Yanzhen Yang, Qu Xie, Chuankai Shang, Lai Jiang, Guojun Ding, Dan Long, Cong Luo

**Affiliations:** ^1^ Postgraduate training base Alliance of Wenzhou Medical University (Zhejiang Cancer Hospital), Hangzhou, Zhejiang, China; ^2^ Department of Hepato-Pancreato-Biliary & Gastric Medical Oncology, Zhejiang Cancer Hospital, Hangzhou, Zhejiang, China

**Keywords:** PDAC, surufatinib, retrospective study, ICI, tyrosine kinase inhibitor

## Abstract

**Background:**

Pancreatic ductal adenocarcinoma (PDAC) is a highly aggressive tumor with a poor prognosis, despite the emergence of chemotherapies such as gemcitabine plus albumin-bound paclitaxel (nab-paclitaxel, AG), unmet medical needs still exist for patients with metastatic PDAC (mPDAC). Surufatinib is a small-molecule tyrosine kinase inhibitor targets vascular endothelial growth factor (VEGFR) 1, 2, 3, fibroblast growth factor receptor 1 (FGFR1), and colony stimulating factor 1 receptor (CSF-1R). This single-center, retrospective study evaluates the potential efficacy of combination therapy containing Surufatinib in advanced or metastatic pancreatic cancer.

**Method:**

We conducted a real world retrospective study of mPDAC patients who received the Surufatinib between July 2022 and July 2023 at Zhejiang Cancer Hospital. In addition, patients who received first line chemotherapy at the same period were analyzed as comparison.

**Result:**

As of November 20, 2024, 20 eligible patients were identified in this retrospective study. The median progression-free survival (mPFS) of patients who received Surufatinib treatment was 5.27 months (95% CI, 2.55–7.98), and the median overall survival(mOS) was 9.93 months (95% CI,6.55-13.32). For fist line treatment, 9 patients received Surufatinib combined with immune checkpoint inhibitors (ICIs) and chemo and the mPFS was 7.5 months (95% CI, 3.14–11.85), compared with an mPFS of 5.43 months (95% CI, 3.89-6.96) for 52 mPDAC patients received chemotherapy at the same period. Grade 3 or above Treatment Related Adverse Event (TRAE) were neutrophil count decreased (10%), and white blood cell count decreased (5%).

**Conclusion:**

Preliminary data suggest that surufatinib shows potential therapeutic benefit in mPDAC, but its efficacy needs to be further validated. This combination strategy may provide a new treatment option for patients, especially in the first-line setting. Future studies will expand the sample size and include additional evaluation parameters to fully assess its efficacy and safety.

**Clinical trial registration:**

ClinicalTrials, identifier NCT06378580

## 1 Background

Pancreatic carcinoma (PC) is a highly aggressive tumor with a poor prognosis, ranking as the 6^th^ leading cause of cancer-related deaths in 2022 (1). According to the 2022 Global Cancer Statistics, there were 510,566 new cases and 467,005 deaths worldwide due to PC. Pancreatic ductal adenocarcinoma (PDAC) accounts for nearly 90% of all PC cases (2). Due to its insidious onset, a large proportion of patients are initially diagnosed with locally advanced or metastatic PDAC, with a 5-year survival rate of only 9% (3). Despite the emergence of chemotherapies such as gemcitabine plus albumin-bound paclitaxel (nab-paclitaxel, AG) or FOLFIRINOX, which have significantly improved survival benefits for patients with advanced PDAC (4–6), unmet medical needs still exist for patients with metastatic PDAC (mPDAC).

Surufatinib is a small-molecule tyrosine kinase inhibitor that targets vascular endothelial growth factor (VEGFR) 1, 2, 3, fibroblast growth factor receptor 1 (FGFR1), and colony stimulating factor 1 receptor (CSF-1R) (7). It was approved in China for patients with neuroendocrine tumors (NETs) based on two pivotal phase III trials SANET-p and SANET-ep. In the SANET-p trial, Surufatinib significantly improved the median PFS of patients with pancreatic NETs compared with the control group (10.9 vs. 3.7 months, HR 0.49, p=0.0011), with an objective response rate (ORR) and disease control rate (DCR) of 19.2% and 80.8%, respectively (8). In the SANET-ep trial, the median PFS was 9.2 vs. 3.8 months for the Surufatinib and placebo control groups, respectively (HR 0.334, P <0.0001), with an ORR and DCR of 10.3% and 86.5%, respectively (9). In addition to pancreatic and extra-pancreatic NETs, early clinical studies of Surufatinib have demonstrated its anti-tumor activity in various tumor types (7, 10).

In our clinical practice, we have observed a certain effectiveness of Surufatinib in combination with PD-1 antibodies in PDAC. Hence, we retrospectively analyzed the real-world efficacy and safety of Surufatinib in metastatic PDAC.

## 2 Methods

### 2.1 Study design and eligibility

This is a real-world retrospective clinical study of patients with mPDAC who received the Surufatinib regimen treatment between July 2022 and July 2023 at Zhejiang Cancer Hospital. Eligible patients met the following criteria: (i) were over 18 years old; (ii) had a histological diagnosis of pancreatic ductal adenocarcinoma; (iii) had at least one measurable lesion; (iv) had undergone at least two cycles of Surufatinib treatment. We also retrospectively analyzed the first-line chemotherapy of patients with mPDAC as the same criteria above mentioned from July 2022 to July 2023 as a comparison.

This study adhered to the ethical standards of the Ethics Committee of Zhejiang Cancer Hospital (the approval number IRB-2024-529) and the Declaration of Helsinki. Written informed consent was waived due to the retrospective nature of the study.

### 2.2 Evaluation, effectiveness, and survival outcomes

All patients in this study were followed up until October 29^th^, 2024. Tumor responses were assessed by physicians every 4–8 weeks using enhanced computed tomography (CT) and/or magnetic resonance imaging (MRI) according to the Response Evaluation Criteria in Solid Tumors (RECIST) version 1.1. The primary endpoint of this study was median progression-free survival (PFS), and the secondary endpoints were median overall survival (OS), objective response rate (ORR) and disease control rate (DCR). PFS was defined as the period from the start of Surufatinib treatment to disease progression. OS was defined as the period from the start of Surufatinib treatment to death from any cause. ORR was calculated as the sum of the proportion of patients with complete response (CR) and partial response (PR). DCR was calculated as the sum of the proportion of patients with CR, PR, and stable disease (SD).

### 2.3 Statistical analysis

Statistical analysis was performed using SPSS software version 26 (IBM, NC, USA). Continuous data were expressed as median, and categorical data were expressed as frequency (percentage). Kaplan–Meier survival curves and the log-rank test were used to compare PFS between different treatment groups. Statistical significance was set at P<0.05.

## 3 Results

### 3.1 Baseline characteristics

**Figure 1:**
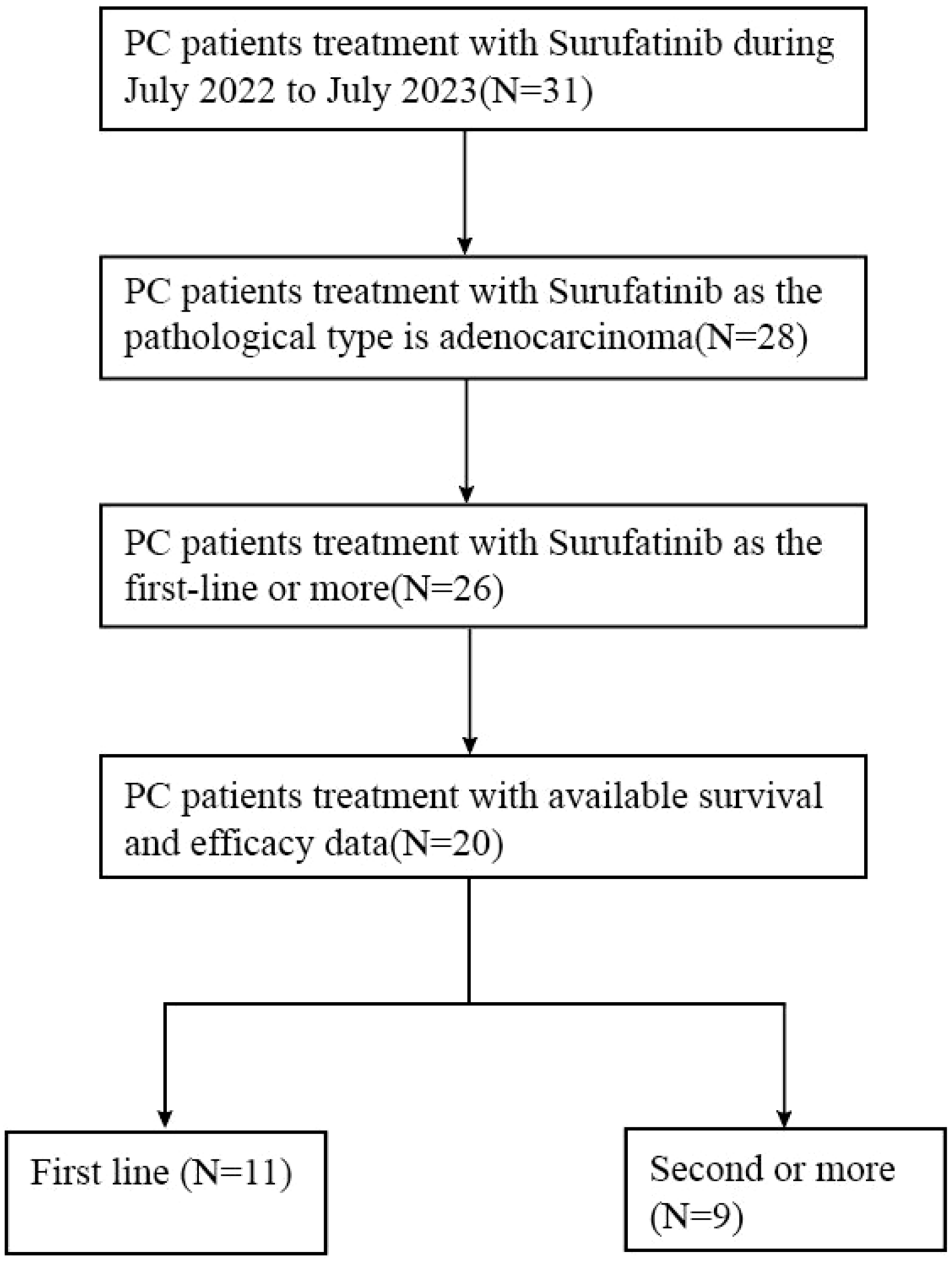
Retrospective study patient disposition.

**Figure 1** illustrates the flow of patient selection. Out of 31 pancreatic cancer patients who received Surufatinib treatment, data from 20 patients were available for retrospective review after screening based on physiological type and follow-up data.

Presents the baseline characteristics of the 20 patients with mPDAC included in this study in **Table 1**. Eleven patients received Surufatinib as first-line treatment, nine received it as second or subsequent line treatment. The median age was 65 years old (range 34-80), with 13 patients (65%) being male. The majority of patients (18, 90%) had an Eastern Cooperative Oncology Group Performance Status (ECOG PS) of 0–1 at the start of Surufatinib treatment, while 2 patients (10%) had a PS of 2 or 3. Six patients (30%) had tumors located in the pancreas head and neck, and 11 patients (55%) had tumors in the body and tail. Eight patients (40%) had undergone surgery before, and one patient (5%) had received radiotherapy before Surufatinib treatment. The most common site of metastasis was the liver, with half of the patients (10) having liver metastasis, 4 patients (20%) having lung metastasis, and 8 patients (40%) having peritoneal metastasis.

**Table 1:** Baseline characteristics of patients with mPDAC.

Characteristics	n	%
Sex		
Male	13	65.0
Female	7	35.0
Age		
Median(years, range)	65.0 (34-80)	
<65	10	50.0
≥65	10	50.0
BMI		
<18.5	1	5.0
18.5-23.9	18	90.0
24-27.9	1	5.0
ECOG		
0-1	18	90.0
2-3	2	10.0
Primary tumor location		
Head and neck	6	30.0
Body and tail	11	55.0
Unknown	3	15.0
Previous treatment		
surgery	8	40.0
Radiotherapy	1	5.0
Number of metastasis site		
Oligo	7	30.0
Multiple	13	60.0
Metastasis		
Liver	10	50.0
Lung	4	20.0
Peritoneal	8	40.0
Other	5	25.0

The summarizes the baseline characteristics of Surufatinib treatment in **Table 2**. Eleven patients (55.0%) received Surufatinib as first-line therapy, while 9 patients (45.0%) received it as second or subsequent line treatment. Among the patients receiving Surufatinib as first line treatment, 9 (45.0%) received Surufatinib combined with immunotherapy and chemotherapy, 2 (10.0%) received it in combination with immunotherapy. Among those who received Surufatinib as second line treatment, 4 patients (20.0%) received Surufatinib in combination with immunotherapy, 3 (15.0%) received it in combination with chemotherapy, 2 (10.0%) received it as monotherapy.

**Table 2:** Baseline characteristics of surufatinib for treatment of mPDAC.

Characteristics of different treatment settings	N	%
First line regimen	11	55.0
Surufatinib + ICI + chemo	9	45.0
Surufatinib + chemo	2	10.0
Second line regimen	9	45.0
Surufatinib + ICI	4	20.0
Surufatinib + chemo	3	15.0
Surufatinib monotherapy	2	10.0

### 3.2 Treatment

Patients included in this real-world retrospective study analysis received Surufatinib at a dose of 200-300mg once daily, orally taken in a consecutive 4-week treatment cycle until disease progression or intolerable toxicity. Some of the patients received Surufatinib in combination with other therapies, including ICI and or chemotherapy. The ICIs that combined with Surufatinib in this retrospective study included PD-1 antibodies as well as a PD-1 and CTLA-4 bi-specific antibody Cadonilimab. The PD-1 antibodies were administered intravenously every 3 weeks. Cadonilimab was administered at a dosage of 6mg/kg intravenously every 2 weeks. All ICIs were administered to patients until disease progression or intolerable toxicity. Chemotherapy regimens that were combined with Surufatinib included AG, S-1, gemcitabine and GEMOX regimens. All these chemo regimens required completion of 6 cycles or until disease progression or unacceptable toxicity. The choice of combination therapy was based on clinical evidence, mechanistic synergies, and patient characteristics; PD-1 monoclonal antibodies were preferred because of their regulatory role in the tumor microenvironment and favorable safety profile (11, 12). Cadonilimab, a PD-1 and CTLA-4 bispecific antibody, activates the anti-tumor immune response more comprehensively (13, 14). Chemotherapeutic regimens (e.g., AG, S-1, gemcitabine monotherapy, and GEMOX) were chosen based on their efficacy, safety, and synergy with surufatinib (5, 15–17).

### 3.3 Treatment response and survival analyses

#### 3.3.1 Response and survival of overall patients

**Figure 2:**
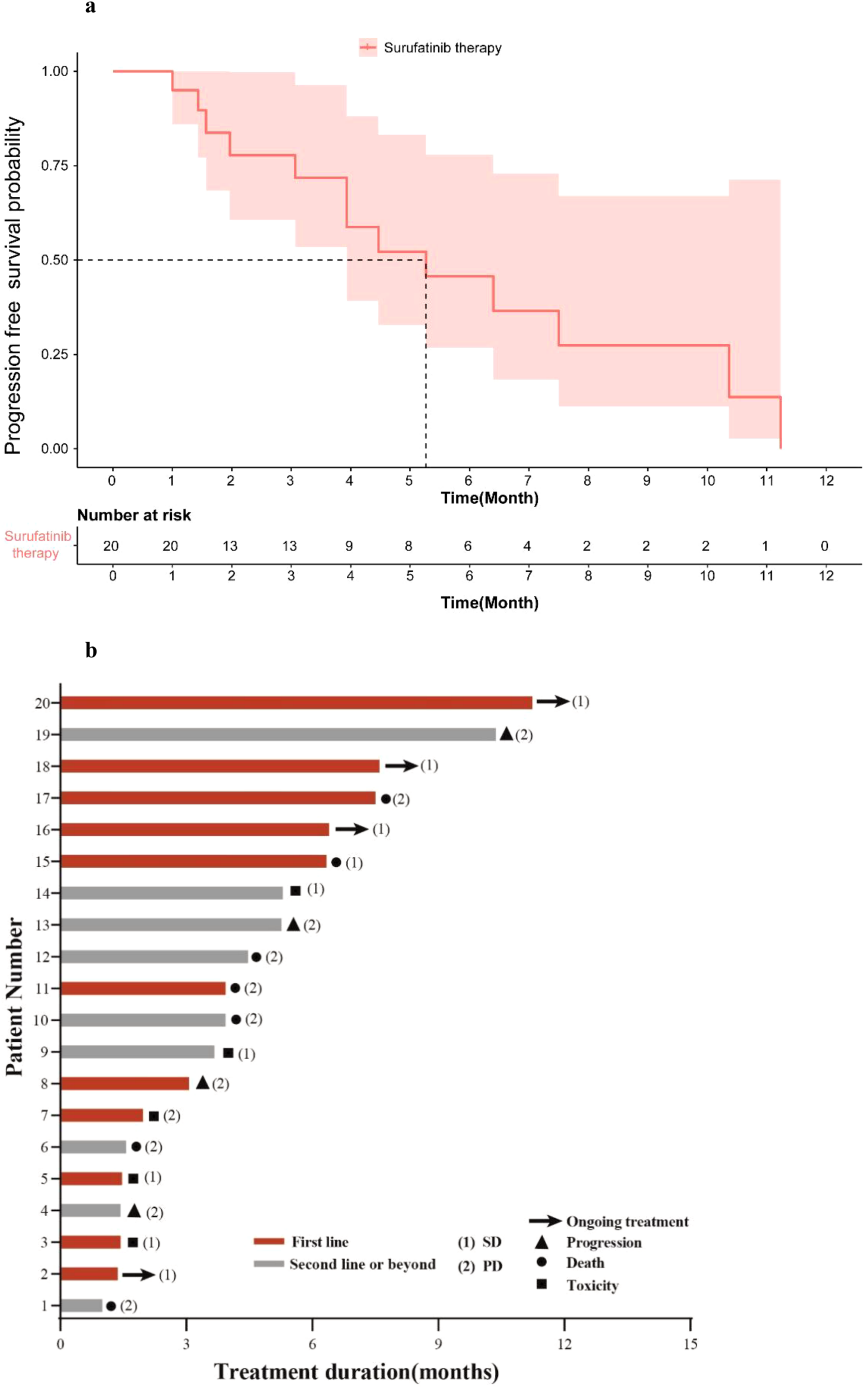
Treatment of Surufatinib in all patients. **(a)** Kaplan-Meier curves for PFS according to the treatment of Surufatinib in all patients. **(b)** Swimming plot of treatment outcomes of patients receiving Surufatinib.

As of Nov.20^th^, 2024, the median follow-up time was 7.08 months, and the mPFS of patients who received Surufatinib treatment was 5.27 months (95% CI: 2.55–7.98) (**Figure 2a**). Treatment of all 20 mPDAC patients with Surufatinib resulted in a disease control rate (DCR) of 35.0% (7/20) (**Table 3**). A swimmer plot illustrating the treatment outcomes of patients receiving Surufatinib was depicted in **Figure 2b**.

**Table 3:** Progression-free survival of treatments summary.

Group	N	Median(months)	95% CI	HR	95%CI	P
**Surufatinib**	20	5.27	2.55–7.98	**-**		**-**
Surufatinib in different treatment lines						
1 L	11	6.4	2.28-10.57	0.337	0.098-1.166	.213
≥2 L	9	4.46	0.76-8.17			
Different treatment in first Line settings						
Surufatinib + ICI + chemo	9	7.50	3.14–11.85	0.485	0.168-1.397	.24
Chemo	52	5.43	3.89-6.96			

#### 3.3.2 Response and survival of surufatinib in different treatment lines

**Figure 3:**
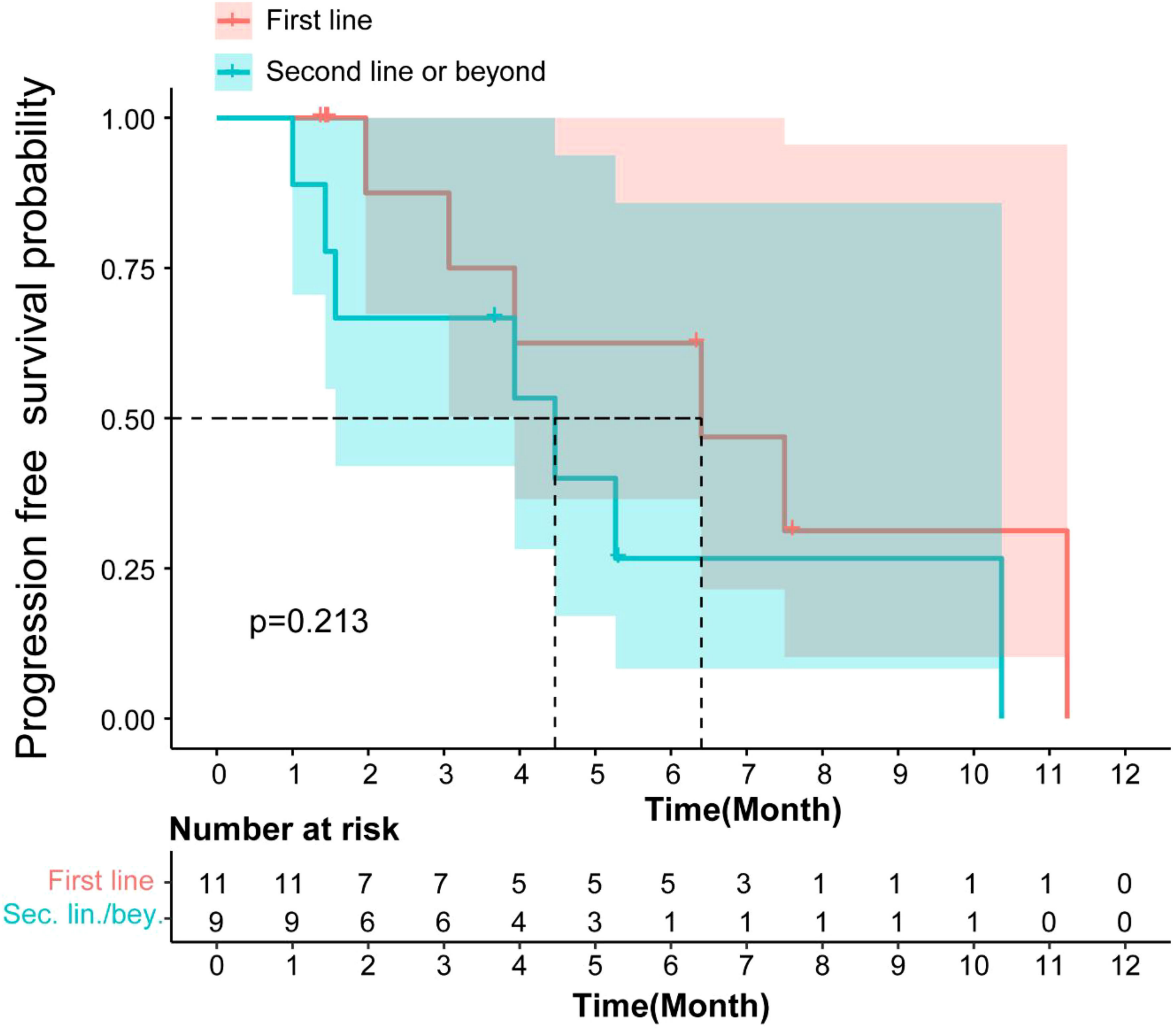
Kaplan-Meier curves for PFS of patients receiving Surufatinib as 1L vs. ≥2L treatment.

The mPFS for patients receiving Surufatinib as first-line treatment was 6.4 months (95% CI: 2.28–10.57), and for those receiving it as second or subsequent line treatment, it was 4.46 months (95% CI: 0.76–8.17). There was no significant difference in mPFS (p=0.213, **Figure 3**) between the first-line and second or subsequent-line treatment groups. The mOS for patients receiving Surufatinib as first-line or second and above line treatment was 10.97 months (95% CI: 5.86–16.08) and 7.17 (95% CI, 2.97–11.36) respectively.

#### 3.3.3 Response and survival of surufatinib combined with ICI and chemo in first line

Furthermore, we analyzed the effectiveness of Surufatinib combined with ICI and chemo in first-line mPDAC patients. Nine patients who received Surufatinib combined with ICI and chemo were identified, and as comparison, 52 mPDAC patients who received chemotherapy in our hospital as first-line treatment were identified. Those who received first-line chemo regimens included AG or FOLFIRINOX. AG regimen consisted of nab-paclitaxel (125 mg/m^2^) followed by gemcitabine (1000 mg/m^2^) on days 1 and 8 every 3 weeks. FOLFIRINOX regimen included oxaliplatin (85 mg/m^2^), irinotecan (180 mg/m^2^), leucovorin (400 mg/m^2^), and 5-fluorouracil (400 mg/m^2^ bolus, 2400 mg/m^2^ continuous intravenous infusion for 46 h) every 14 days. Both the AG regimen and FOLFIRINOX regimen in the first line needed to complete 6 cycles or until the progression of the disease or unacceptable toxicity.

The baseline characteristics of Surufatinib combined with ICI and chemo of first-line treatment were displayed in **Table 4**. All patients had an ECOG PS of 0–1 in the Surufatinib combined with ICI group, whereas there were 48 (92.31%, 48/52) patients with an ECOG PS 0–1 in the chemotherapy group. The liver is the most common site of metastases in the Surufatinib combined with ICI and chemo group, while the lung is the most common site of metastases in the chemotherapy group.

**Table 4:** Baseline characteristics of patients with different treatment in 1L.

Items	Surufatinib+ICI N=9	Chemo N=52	P
Sex			
Male	7	36	
Female	2	16	
Age			
<65	3	23	
≥65	6	29	
BMI			
<18.5	0	7	
18.5-23.9	8	32	
24-27.9	1	13	
ECOG			
0-1	9	48	
2-3	0	4	
Local of tumor			
Head and neck	2	14	
Body and tail	6	35	
Unknown	1	3	
Previous surgery			
Yes	3	2	
No	6	50	
Number of metastasis site			
Oligo	2	2	
Multiple	7	50	
Metastasis			
Liver	4	36	
Lung	0	41	
Peritoneal	3	25	
Other	5	41	

**Figure 4:**
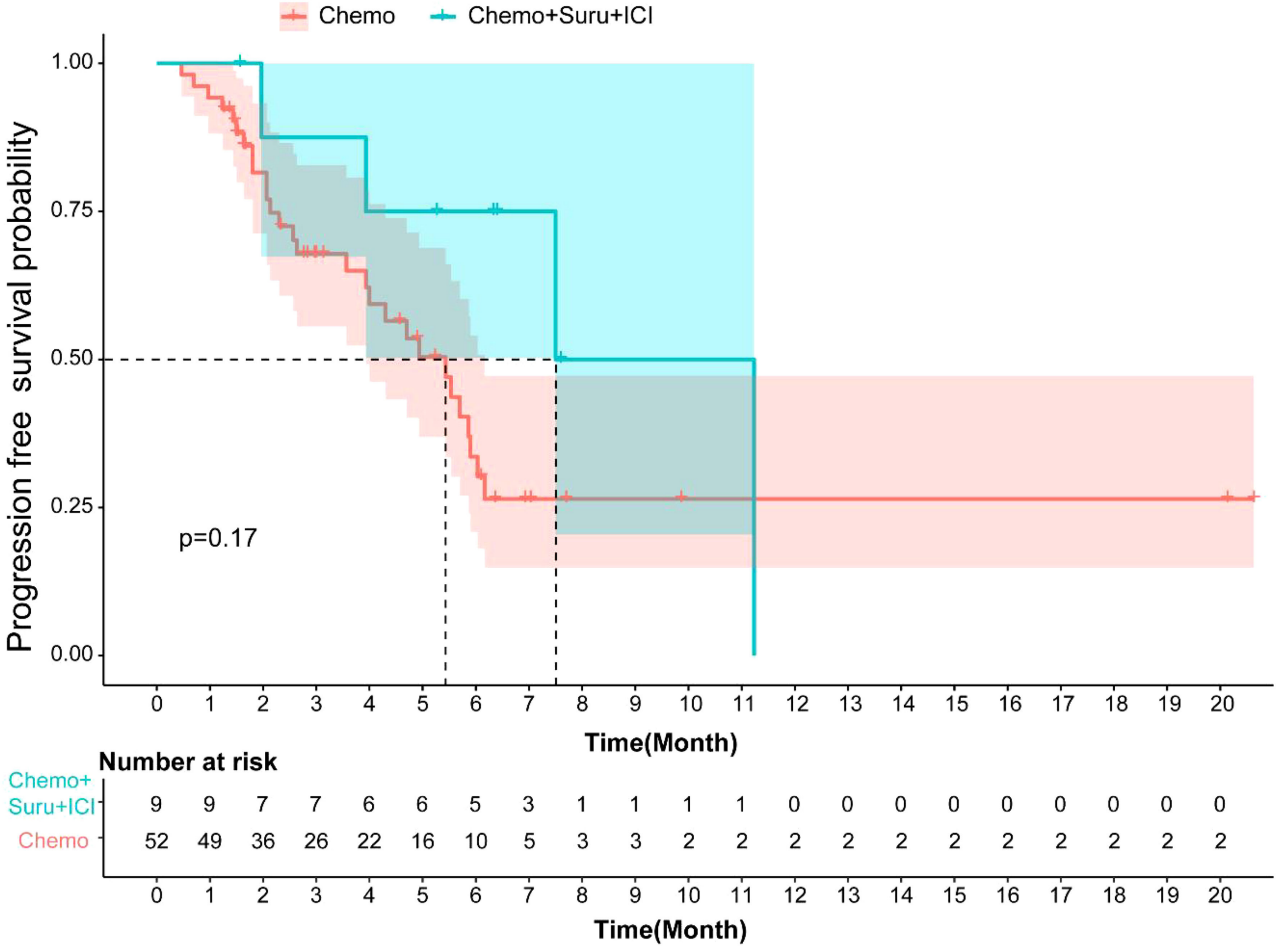
Kaplan-Meier curves for PFS of patients with different treatment regimens in first line.

The median PFS for patients in the Surufatinib combined with ICI and chemo group was 7.5 months (95% CI, 3.14–11.85), while it was 5.43 months (95% CI, 3.89-6.96) in the chemotherapy group. There was no significant difference in mPFS (p=0.17, **Figure 4**) between these two treatment groups. The DCR of the Surufatinib combined with ICI and chemo group was 55.56% and the DCR of chemotherapy was 44.23%, with no significant difference in DCR between these two groups (p=0.772).

### 3.4 Safety

All patients underwent safety assessment, including physical examination, laboratory tests, and imaging tests, every 2 weeks during the treatment period. Adverse events were documented through the electronic medical record system and categorized and graded using CTCAE version 5.0. The relevance of adverse events to treatment was assessed by the study team, and appropriate management measures were taken according to severity. The treatment-related adverse events (TRAE) of any grade in all 20 mPDAC patients are summarized in **Table 5**. The most common (≥10%) any grade TRAEs were red blood cell count decreased(25%), platelet count decreased (25%), white blood cell count decreased(20%), ALT increased (20%), AST increased(15%), vomiting (10%). ≥Grade 3 TRAE were neutrophil count decreased (10%), and white blood cell count decreased(5%).

**Table 5:** Treatment related adverse events of patients received Surufatinib.

Primary Term	Any Grade	≥Grade 3
Red blood cell count decreased	5 (25%)	0
Platelet count decreased	5 (25%)	0
White blood cell count decreased	4 (20%)	1 (5%)
Alanine aminotransferase increased	4 (20%)	0
Aspartate aminotransferase increased	3 (15%)	0
Vomiting	2 (10%)	0
Neutrophil count decreased	1 (5%)	2 (10%)
Proteinuria	1 (5%)	0

## 4 Discussion

Pancreatic cancer is a highly malignant tumor with a poor prognosis, characterized by insidious onset, rapid progression, and short survival time. It is considered one of the malignancies with the worst prognosis (18). The standard first-line treatments for mPDAC are the modified FOLFIRINOX regimen or the AG regimen (4–6). while in real-world Gemcitabine may also being used, as metastatic PDAC patients might often have compromised physical conditions. In our study, patients who received chemotherapy as first-line treatment in our hospital had a median PFS of 5.43(95% CI, 3.89-6.96) months, consistent with previous reported real world data, mPFS ranged from 3.33 to 5.37 months, mOS ranged from to 8.5 to 10.0 months (19, 20). For patients in the second line or later, regimens different from those used in the first line are suggested. Previous studies have reported mPFS of 2.51 months for gemcitabine alone and 3.61 months for nab-paclitaxel plus gemcitabine in mPDAC patients who progressed after first-line FOLFIRINOX (21).

Surufatinib has demonstrated its potential efficacy in pancreatic cancer: A preclinical study using a transplantation mice model of pancreatic cancer and a co-culture system of pancreatic cancer cells and tumor-associated macrophages (TAMs) suggested that Surufatinib may enhance the efficacy of AG and PD-1 antibody by mitigating AG-induced immunosuppression and resistance (22). Two clinical trials both use Surufatinib combined with PD-1 antibody and chemo regimens demonstrated the potential efficacies in first line mPDAC treatment (23, 24). Surufatinib plus TAS102 was also effective in patients with mPDAC who had received at least two prior lines of therapy (25). With the preliminary exploration of regimens containing Surufatinib in mPDAC, our real-world experiences of Surufatinib was deemed as demonstrative supporting data in its efficacy towards mPDAC.

Real-world studies differ from randomized controlled trials (RCTs) in that treatment options are selected based on actual conditions and patient preferences. In this real-world retrospective study, all patients provided informed consent for their tumor treatment. Prior to treatment, patients perceived the efficacy of existing standard treatments as limited. Therefore, in pursuit of better outcomes, they chose treatment regimens that included Surufatinib.

In our real-world retrospective study, patients achieved mPFS of 5.27 months and mOS of 9.93 months with Surufatinib therapy, including both first-line and second-line or later patients. For first-line mPDAC, the mPFS was 6.40 months, and when Surufatinib combined with immunotherapy and chemotherapy, the mPFS was 7.50 months, the mOS were 10.97 months. For patients in the second line or later, the mPFS and mOS were 4.46 months and 7.17 months, similar to the data reported in the German CONKO-study group (4.82 months and 9.09 months respectively) (26). Previous studies on anti-VEGFR or immunotherapy alone in mPDAC have not demonstrated satisfactory results (27, 28). The combination of anti-VEGFR TKI and immunotherapy and chemotherapy in our study showed significant survival benefits, comparable to cytotoxic chemotherapy, in unselected mPDAC patients.

We compared the effectiveness of Surufatinib plus immune checkpoint inhibitors plus chemo with chemotherapy in the first-line treatment of metastatic pancreatic ductal adenocarcinoma. The median progression-free survival for patients who received Surufatinib combined with ICI and chemo was 7.5 months (95% CI, 0.90-14.09), while it was 5.43 (95% CI, 3.89-6.96) months for patients who received first-line chemotherapy in our hospital. Although there was no significant difference in PFS (p=0.17) between these two groups, Surufatinib combined with ICI and chemo showed a trend towards better survival benefits in the first-line setting. Compared with clinical trial data, the mPFS of surufatinib in combination with ICI and chemotherapy in this study (7.5 months) was slightly lower than that of surufatinib monotherapy for neuroendocrine tumors (10.9-15.2 months) (9, 29), but higher than that of immunocombination chemotherapy for advanced pancreatic cancer (2.78 months) (30). The combination regimen in this study showed longer mPFS (7.5 months vs. 3.33-5.37 months) compared to real-world data (19, 20), suggesting a potential benefit of surufatinib in combination with ICI and chemotherapy in PDAC.

Because this was a retrospective study and relevant time-point sampling was not retained in time, mechanism validation is lacking in this paper. By searching data from published literature in recent years, we organized the possible mechanisms of benefit as follows, with the expectation that they will be validated in future prospective studies. Surufatinib provides a biological basis for the synergistic effect of ICI and chemotherapeutic agents by inhibiting VEGFR, FGFR, and CSF-1R, exerting anti-angiogenesis and regulating the tumor microenvironment (7, 12). ICI enhances the immune system’s effect on tumor killing by restoring the T cells’ anti-tumor activity and enhances the killing effect of the immune system on tumors (31–33). Chemotherapeutic agents further enhance the anti-tumor immune response by inducing apoptosis and immunogenic cell death (ICD) in tumor cells (34, 35). The synergistic effect of combination therapy may be realized by improving the immune microenvironment, enhancing T-cell infiltration and inducing immunogenic cell death, thus significantly improving the therapeutic effect.

The safety profile of Surufatinib combined with ICIs was consistently acceptable, as shown in previous trials (15–17). The combined regimen of Surufatinib and ICI and chemo were also acceptable indicated by previous studies (12, 13). Based on the available information, the treatment regimens were expected to be safe in clinical settings, which enhanced our confidence in their use. In our real-world retrospective study, no unexpected safety signals were identified, and most treatment-related adverse events were grade 1 or 2. The most frequently observed TRAEs were red blood cell count decreased (25%), platelet count decreased (25%). However, the safety outcomes in real-world studies primarily rely on patient self-reporting. If patients experience adverse events such as proteinuria, which is insidious and can only be detected through urine tests, as well as diarrhea and hypertension, which they may find tolerable and choose not to report, the collected information is often less comprehensive than that in RCTs. Consequently, the incidence of AEs reported in real-world studies is typically lower than that reported in RCTs.

However, there are several limitations to this present study. It is a single-center real-world retrospective study with a relatively small sample size, heterogeneity in the treatment is a major limitation of the study. However, as the nature of real-world, the selection of treatment measures was based on the actual conditions and preferences of patients. We aim to evaluate the regimens containing Surufatinib in real world settings, we did not make restrictions on the specific regimens. Based on medical records, 20 patients treated with regimens containing Surufatinib was tracked. In this study, due to the diversity of the patients’ underlying conditions, some opted for Surufatinib combined with ICI, while others chose Surufatinib combined with chemotherapy. Additionally, two patients chose Surufatinib monotherapy. As a real-world study, the patient treatment information collected in this study did not specify the details of ICI. Additionally, due to the intolerance of the Chinese population, the actual use of the AG regimen in China differs from the phase III study protocol, which was conducted mainly in Europe and North America, with a common practice being a three-week regimen on days 1 and 8, instead of a four-week regime on days 1, 8 and 15. Furthermore, the results need further confirmed by large-sample prospective studies.

## 5 Conclusion

The anti-tumor activity of Surufatinib in mPDAC patients is promising. First-line use of Surufatinib can achieve better efficacy compared to second or later lines of treatment. Surufatinib combined with immunotherapy may further improve the efficacy in mPDAC and provide a potential treatment option for patients, especially in the first-line setting.

## Data Availability

The datasets generated during and/or analyzed during the current study are available from the corresponding author on reasonable request.
